# Effects of appearance modifications on oral presentation anxiety in video conferencing

**DOI:** 10.1038/s41598-025-15716-z

**Published:** 2025-08-17

**Authors:** Ziting Gong, Hideaki Kanai

**Affiliations:** Graduate School of Advanced Science and Technology, Japan Advanced Institute of Science and Technology, 9231292 Ishikawa, Japan

**Keywords:** Oral presentation anxiety, Video conferencing, Facial filter, Psychological state, Physiological signal, Psychology, Human behaviour

## Abstract

Oral presentations are considered to provoke high levels of anxiety, yet limited research has explored this phenomenon in the context of video conferences. Considering the feasibility of private facial filters in video calls and the impact of audience appearance on speakers’ experiences, we propose modifying the visual representation of the audience to reduce oral presentation anxiety in video conferences. We found literature evidence supporting the effectiveness of both highly familiar appearances and attractive anime characters in reducing anxiety. Building on this, we conducted a real video presentation experiment with 45 participants, comparing the effects of these two visual modifications through physiological signals, behavioral data, and self-reported measures. The results showed that the appearance of a good friend as a facial filter more effectively alleviated self-perceived oral presentation anxiety and led to higher performance evaluations during one-on-one video conferences, confirming that a familiar audience is a more universally effective approach. On the other hand, the anime character primarily reduced the speaker’s attention to the audience’s gaze, potentially benefiting in reducing the pressure of being watched. By shedding light on the role of audience appearance in video conference anxiety, this study offers valuable insights and practical strategies for designing customized computer-mediated communication environments that enhance speaker comfort and confidence.

## Introduction

Anxiety is generally considered a feeling of worry, unease, or apprehension about an uncertain outcome^1^. From a psychological point of view^2^, it is considered an emotional state manifested through sensations of tension, worrying thoughts, and physical changes^3^. According to cognitive-behavioral theory^4^, anxiety often stems from negative thoughts and irrational beliefs. The anxiety sensitivity theory^5^ suggests that anticipatory anxiety occurs before an event, as individuals fear the potential occurrence of negative outcomes. Meanwhile, the evolutionary paradigm^6^ views anxiety as an adaptive response that helps enhance alertness when facing challenging environments. Anxiety permeates various aspects of our daily lives, including communication anxiety, test anxiety^3^, programming anxiety^7^, math anxiety^8,9^, computer anxiety^10^, among others.

Oral presentations have long been regarded as one of the most anxiety-inducing events^11–14^. Given that prolonged anxiety can lead to various adverse effects, such as limitations in personal social interactions, erosion of self-esteem and confidence, and negative impacts on work performance^7,8,15,16^, various measures have been proposed to alleviate such anxiety. For instance, previous studies have leveraged virtual reality (VR) to help them overcome presentation anxiety by being repeatedly exposed to public speaking scenarios^17–22^. Additionally, one study explored the usage of speech-based feedback to address anxiety. It first recorded the speaker’s voice under stable emotional conditions to capture its characteristic features. During the presentation, the speaker receives the modified voice which reflects the characteristics of their stable-state voice, effectively creating the illusion that they were composed and confident aiming to reduce the speaker’s anxiety^23^.

Oral presentations are typically considered to take place in face-to-face settings. However, with the rapid development of computer-mediated communication technology in recent years, giving presentations through video conferences has become very common. However, there is limited research on the anxiety experienced by speakers in video conferences which is based on computer-mediated communication platforms.

In our investigation of video conferences, we found that although it has revolutionized communication by providing a platform for efficient and instantaneous connections across various settings, this technology also presents challenges, including video conferencing fatigue, which is perceived as more mentally taxing than face-to-face interactions^24^. Previous studies have suggested that heightened anxiety levels during video conferences may stem from platform characteristics, particularly the sense of close proximity^25,26^. The intimacy created by the proximity between users and screens can make people feel uncomfortable when interacting with unfamiliar individuals, thereby increasing anxiety. According to interpersonal distance norms, the personal distance for interactions among close friends or family falls within a close range of 0.46 to 0.76 meters. Maintaining a social distance of 1.2 to 2.1 meters is generally considered more comfortable when interacting with strangers^27^. However, during video conferences, the physical distance between participants and the unfamiliar faces on the screen typically shrinks to about 0.5 meters, leading to discomfort and anxiety due to the significant deviation from expected social norms.

Additionally, the limited environmental cues in video conferencing settings amplify the prominence of participants’ gazes^28–31^. In traditional face-to-face conferencing scenarios, various environmental factors and body language cues help distribute attention and reduce the intensity of direct eye contact. However, in video conferencing scenarios, the absence of these contextual elements makes participants’ gazes more pronounced. This increased eye contact not only creates discomfort from being constantly observed but also leads to the anxiety of maintaining it, thus causing anxiety^32^. In summary, performing a presentation in video conferences triggers inherent anxiety not only from the event itself but also due to the platform of video conferences.

Video conferences offer a distinct advantage of modifying visual contents on the screen that may influence our perception. Virtual background selection plays a vital role in shaping others’ perceptions. Previous research revealed that concealing the original setting using virtual backgrounds effectively mitigated extreme ratings of speakers’ personality traits^26^. A commonly recommended strategy for reducing anxiety during oral presentations is to mentally reframe the audience in a less intimidating and more familiar context. For instance, speakers are often advised to imagine their audience in humorous or mundane situations—such as picturing them wearing mismatched socks or having holes in their socks—to reduce the perceived threat of negative evaluation^33–35^. Such mental reframing techniques aim to foster a more relaxed internal environment by lowering the audience’s perceived authority or formality. Extending this idea into the digital domain, computer-mediated communication platforms like video conferencing now offer technological means—such as Augmented Reality (AR) filters—to visually transform the audience’s appearance. These filters provide an opportunity to create a presentation context that is less intimidating and more psychologically comfortable. Specifically, comfort may arise through two complementary pathways: familiarity and visual abstraction. Familiar faces, such as those of close friends, can evoke a sense of trust and safety, reducing anticipatory anxiety by minimizing fear of negative judgment^36^. On the other hand, cartoon-like or anthropomorphic avatars introduce a layer of abstraction that distances the speaker from the realism of social evaluation. These non-threatening, stylized visuals reduce perceived interpersonal intensity, creating an emotionally buffered environment that supports more confident speaking behavior^34,37^. A previous study surveyed 100 participants to investigate their perceptions of this private AR face filter technology^34^. The results revealed a high level of acceptance, even when the filter was privately applied to themselves. One primary reason for using the filter was to reduce negative emotions such as anxiety and fear, thereby creating a more relaxed atmosphere. Additionally, applying filters to others’ faces was often motivated by the desire to diminish the sense of intimidation posed by others.

Building upon these insights and the psychological mechanisms underlying audience perception, the present study investigates whether visually altering the appearance of the audience can help reduce oral presentation anxiety in one-on-one video conferencing settings. Specifically, we examine two types of visual modifications: replacing the audience’s face with that of a close friend (to evoke familiarity), and with an attractive anime character (to introduce visual abstraction). To assess their effects, we conducted a between-subjects experiment with 45 participants, analyzing physiological signals, user behaviors, and self-reported perceptions. This approach aims to provide empirical evidence on how different visual strategies may alleviate presentation-related anxiety and inform the design of supportive communication technologies in virtual settings.

## Face-filter proposal

In addition to audience behavior^38–41^, attitude^39,42^, presence^43^, and size^18,44,45^, the appearance of the audience^14,46^ has also been found to influence the oral presentation anxiety levels in virtual environments. In the context of video conferencing, privately applying AR filters to modify the appearance of the other party on one’s own screen holds great potential for alleviating users’ social anxiety, especially in anxiety-inducing situations such as interviews or public speaking^34,37^.

### Audience familiarity

Audience familiarity is considered an important factor in eliciting anxiety^36,46–49^. Familiar friends as audience are often perceived as more tolerant and understanding, with a lower likelihood of giving negative evaluations^36^. Consequently, speakers are likely to anticipate a pleasant speech environment, allowing them to deliver speeches in a more relaxed manner. When the actual situation aligns with this expectation, familiar friends as audience evoke the lowest levels of tension. However, if the expectation is not met and the audience behaves unpleasantly, familiar audiences may provoke anxiety levels similar to or even greater than those caused by unfamiliar audiences^50,51^. This observation was further supported by MacIntyre and Thivierge’s study^36^, in which participants were asked to imagine giving speeches in front of audiences that varied in familiarity and emotional expressions. Their findings indicated that anxiety was lower when speaking in front of friends, as a high familiarity. However, the effect of familiarity was weaker compared to that of pleasant facial expressions. Future studies should focus on designing experiments that measure actual emotional responses to minimize potential biases arising from imagined scenarios. In particular, it is crucial to control for the influence of emotional feedback when assessing the effect of familiarity on anxiety. Additionally, the study required participants to imagine an audience size of 20, raising some questions, such as 20 distinct or 20 identical faces. This ambiguity could impact the interpretation of familiarity effects. Despite some limitations, this study provides preliminary evidence that highly familiar audiences can be effective in alleviating anxiety during oral presentations.

Previous study^14,46^ examined how familiar and unfamiliar audiences influence speakers’ anxiety levels during foreign language speeches in both VR and real-world settings. In the VR environment, familiar audiences were found to induce higher anxiety compared to unfamiliar ones. The authors attributed this to the lower realism of computer-generated unfamiliar audiences, which might have reduced the sense of presence and, consequently, the anxiety they provoked. In contrast, familiar audiences were represented using scanned 3D images. However, the authors later challenged their explanation by referencing prior literature^43,52^ suggesting that photorealism is not a precursor to the sense of co-presence in virtual environments. In the real-life setting, no significant effect of audience familiarity on anxiety was observed. Additionally, the results indicated that individuals with a moderate fear of public speaking experienced reduced anxiety when presenting in front of virtual audiences composed of familiar faces. The small sample size of the experiment (10 participants) and the choice of familiar faces, such as teachers and researchers, who are often perceived as authoritative figures, who could potentially heighten anxiety^53^, may have influenced the effect of audience familiarity.

To bridge the gap in empirical research on the effects of highly familiar audiences, we propose using the appearance of good friends as a facial filter to alleviate oral presentation anxiety during online conferences. Moreover, the choice is likely to minimize the presence of authoritative audiences, thereby reducing their potential impact on anxiety levels.

**Hypothesis 1: Modifying the interviewer’s appearance to resemble a good friend will reduce the speaker’s anxiety in one-on-one video conferences.**

### Non-human entity

Leong et al.^34^ found that compared to makeup or accessory-based modifications, most participants preferred applying non-human AR filters, such as lions, to the audience’s faces during presentations. These full-face filters completely masked the real human appearance, reducing the sense of realism in the online public speaking environment and thereby alleviating the anxiety of being judged. Additionally, these non-human characters were perceived as cute and friendly, which helped speakers feel more confident and contributed to reducing presentation anxiety during video conferences^34^. While animal appearances can help mitigate the anxiety of negative evaluations, they also introduce additional challenges. One major issue is the lack of facial expression cues, which may lead to uncertainty and discomfort for the speaker. A promising solution to this problem is the use of anthropomorphic characters. Their human-like appearance provides a sense of familiarity and facial expression cues^37^, while their non-human nature can still reduce realism and alleviate the anxiety associated with being judged. This idea is supported by the study conducted by Girondini et al.^54^, which found that delivering a speech in front of real humans induced higher anxiety levels than speaking in front of cartoon characters. Moreover, using anime characters to cover the face of one’s conversation partner is considered similar to positive facial feedback, making interactions feel easier for individuals with social anxiety^55^.

Yoneyama et al.^37^ explored the impact of modifying a conversation partner’s appearance into either an anime character or a real person using AR head-mounted displays (HMD) during real-life interactions. Their experiment found that anime characters did not significantly reduce anxiety during casual interview sessions. This result might be influenced by a practice effect, as participants repeated the experiment across control, avatar, and smile conditions. Furthermore, in the smile condition, the visual appearance remained the same as in the control condition (a real human face), which may have introduced familiarity effects. Additionally, both the control and avatar conditions featured positively biased facial expressions, masking the actual expressions of the communication partner. This may also explain why anxiety levels in the smile condition were also not significantly lower than in the control condition. Although there is no clear evidence that anime characters alleviate anxiety due to their results being mixed with positive facial feedback and facial familiarity, researchers suggest that anime characters create a sense of warmth, enhancing the comfort of interpersonal interactions. Considering their research findings and the advantages of anthropomorphic characters, we are highly confident in the potential of using attractive anime characters as facial filters to alleviate speakers’ anxiety, particularly after minimizing practice effects and the influence of positive facial expressions.

**Hypothesis 2: Modifying the interviewer’s appearance to an attractive anime character will reduce the speaker’s anxiety in one-on-one video conferences.**

## Validation experiment

We compared the effects of the two aforementioned face filters on speakers’ anxiety during presentations in a real video conference scenario. We employed a between-group design to minimize practice effects. The participants were randomly divided into one of three groups (conditions):

- The Good Friend Interviewer (GFI) group, where the interviewer’s appearance is modified to resemble that of the speaker’s good friend.
- The Anime Character Interviewer (ACI) group, where the interviewer’s appearance is modified to resemble that of an attractive anime character.
- The Stranger Interviewer (SI) group, where the interviewer appeared as a default stranger, as the control condition.

### Experiment design

We designed an experiment in which participants delivered a 3-minute presentation in front of an interviewer who used existing software to apply face filters, masking his real appearance. We utilized Avatarify Desk (https://github.com/alievk/avatarify-desktop) to manipulate the appearance of the interviewer as seen by the participants. This software maps real-time facial and head movements onto a subject in a photograph, enabling characters in still images to exhibit natural expressions and bodily movements, closely resembling those in actual video interactions.

We invited a 30-year-old male PhD candidate from another unit to act as our interviewer (practical assistant). Before each experiment, the interviewer conducted thorough tests to ensure that his facial expressions (neutral but slightly negative) and movements were accurately mapped onto the corresponding positions in the photograph. During the presentation task, the assistant was instructed to maintain eye contact with the participant throughout the session, except for 1–2 instances of looking down to simulate note-taking. If a participant finished their presentation early, the assistant would remain in the note-taking posture until the video conference automatically ended.

To better simulate an authentic interview environment, neutral reading tasks and quiet waiting periods were arranged before and after meeting the interviewer. These tasks served two purposes: first, to ensure participants were alone in the room during the video conference, giving the experimenter time to leave and re-enter; and second, to reduce pre-interview anxiety by redirecting participants’ attention to the reading material. The detailed task schedule was as follows:

- 1-minute neutral reading task: Participants were instructed to read the content displayed on the screen, which included experimental instructions and an introduction to the automatic transitions.
- 3-minute presentation task with the interviewer: The screen displayed a live video of the interviewer via Cisco Webex App version 42.6 (https://www.webex.com). To enhance immersion, the small window showing the participant’s video and the menu bar were disabled, allowing the interviewer’s video to occupy the entire screen.
- 30 seconds post-presentation reflection: A prompt displayed the message, “Presentation completed, please wait quietly for the experimenter to return,” simulating a brief period of reflection after the presentation.

The transitions between tasks were implemented by pre-loading the relevant materials in the corresponding sequence and setting them to automatically close at specific times. The detailed settings were as follows:

- Full-screen reading material (top layer): Automatically closed after 60 seconds.
- Adjusted video conferencing software window (middle layer): Automatically closed after 240 seconds.
- Image with concluding text (bottom layer): Automatically closed after 270 seconds.

Based on the prior experiment^56^, the preparation time for the presentation was set at 7 minutes, which was sufficient for participants to outline their main points. The speech topic was assigned as: “Please introduce your strengths and/or weaknesses,” as this is one of the most common interview questions. Participants could choose to focus on either one aspect or both, aiming to reduce potential anxiety from prematurely ending their presentation.

**Fig. 1:**
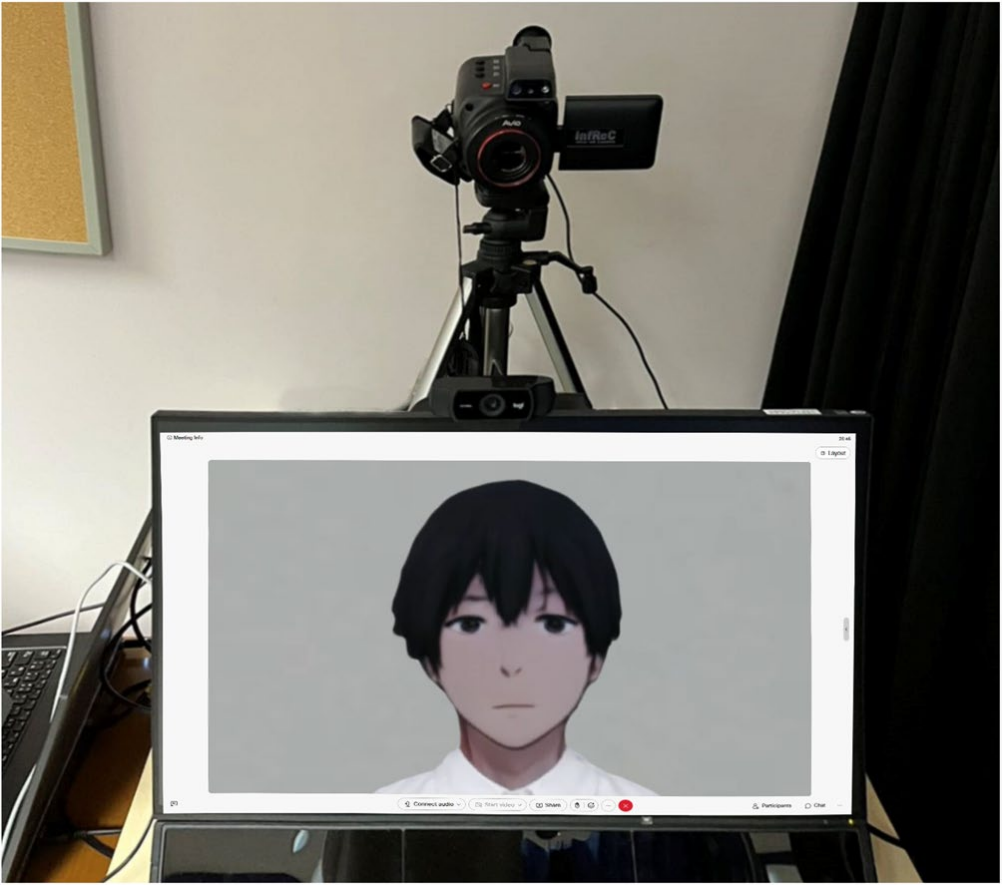
Experimental setup under the Anime Character Interviewer (ACI) condition. An anime character with the highest likability rating was displayed as the interviewer during the presentation.

For the ACI condition, we selected five male anime character images from the internet. These images were evaluated by 20 students based on familiarity, anxiety, and likability. The most highly rated character, which scored highest in likability and familiarity, and lowest in anxiety, was chosen as the ACI interviewer (See Fig. 1). For the GFI condition, participants provided a frontal photo of one of their good friends. For the SI condition, the practical assistant’s own frontal photograph was used as the stranger’s appearance. Thus, in the ACI and SI groups, the audience appearance was predetermined and consisted of a single character or face, whereas in the GFI group, the appearances varied. To standardize the visual presentation across conditions, we pre-adjusted the images to ensure similar head proportions and positioning on the screen. Using Adobe Photoshop, we also ensured uniformity in clothing, head size, and background, maintaining a light grey backdrop for all three conditions. These appearances are illustrated in Fig. 8, showcasing the visual representations of the interviewers across the conditions in eye-tracking results.

**Fig. 2:**
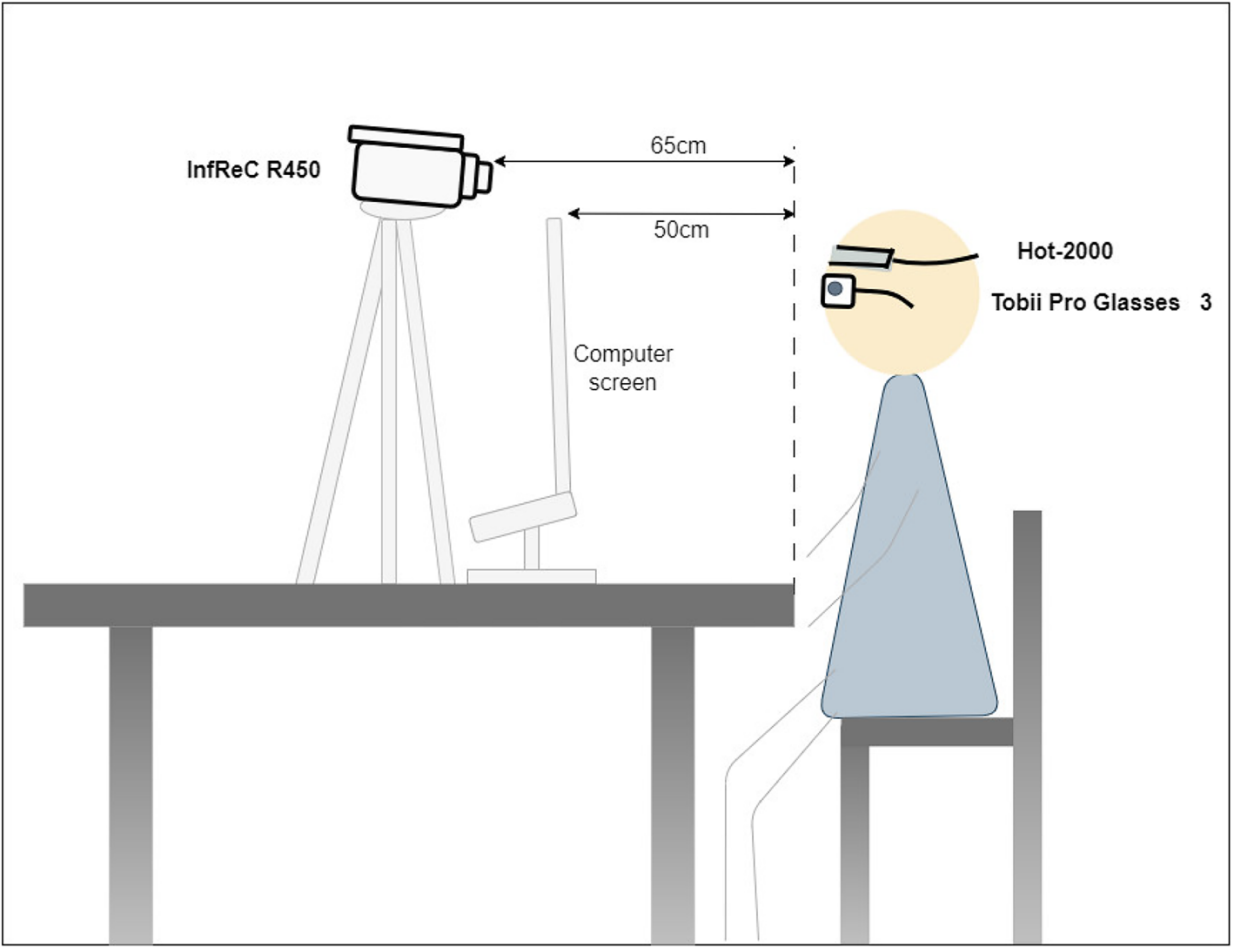
Layout of the experimental room and devices. Shows the participant’s position and measurement devices, including eye-tracking glasses, fNIRS, and thermal imaging.

The experimental room layout is shown in Fig. 2, highlighting the equipment worn by participants, including the eye-tracking device (Tobii Pro Glasses 3) and the functional near-infrared spectroscopy (fNIRS) device (Hot-2000). Additionally, the setup features an infrared thermal imaging camera (InfReC R450). Furthermore, we administered questionnaires, including the State-Trait Anxiety Inventory (STAI), the Perception of Speech Performance Scale (PSP), and a self-designed questionnaire, to collect subjective data.

### Measures

**Eye-tracking** We utilized the Tobii Pro Glasses 3 (Tobii AB, Danderyd, Sweden), a wearable eye-tracking device resembling regular eyewear, designed for precise recording of participants’ eye movements. The system comprises a head unit, a recording unit, and a controller application, which can be installed on a computer for data management. The glasses feature removable lenses tailored to correct near-sightedness and far-sightedness, with lens options ranging from +3 to -8.0 diopters for each eye, adjustable in 0.5 diopter intervals. With a sampling rate of 100 Hz, the device was configured and calibrated for optimal accuracy in tracking eye movements.

The device recorded participants’ eye movements during the experimental tasks. This setup enabled us to analyze participants’ visual behaviors during the presentation task, offering valuable insights into their focus and attentional patterns.

**Cerebral blood flow changes** We utilized the Hot-2000 device (NeU Corp, Tokyo, Japan) to measure changes in cerebral blood flow. The device featured two source-detector (SD) pairs positioned on the left and right sides of the forehead, each consisting of a 1-cm SD pair and a 3-cm SD pair. The device emitted infrared light at a wavelength of approximately 800 nm and recorded data at a sampling rate of 10 Hz. The 3-cm SD pair functioned as a long-separation detector, capturing changes in blood flow linked to neural activity, while the 1-cm SD pair acted as a short-separation detector to filter out interference from the scalp.

Increased activity in the prefrontal cortex (PFC), particularly in the left PFC, has been shown to occur during the regulation of negative stimuli^57,58^. This device enabled us to analyze changes in participants’ cerebral blood flow before and after seeing the interviewer, potentially offering insights into the neural responses elicited by the interviewer’s appearance.

**Thermal infrared imaging** We used thermal imaging with the InfReC R450 device (Nippon Avionics Co., Ltd, Yokohama, Japan) to measure changes in participants’ nasal temperature. This non-contact method allows the detection of emotional states by capturing changes in facial temperature^59–61^. The InfReC R450 recorded facial temperatures at a rate of one frame per second, using a 480 $\times$ 360 detector pixel array with spectral sensitivity ranging from 8 to 14 micrometers.

**Fig. 3:**
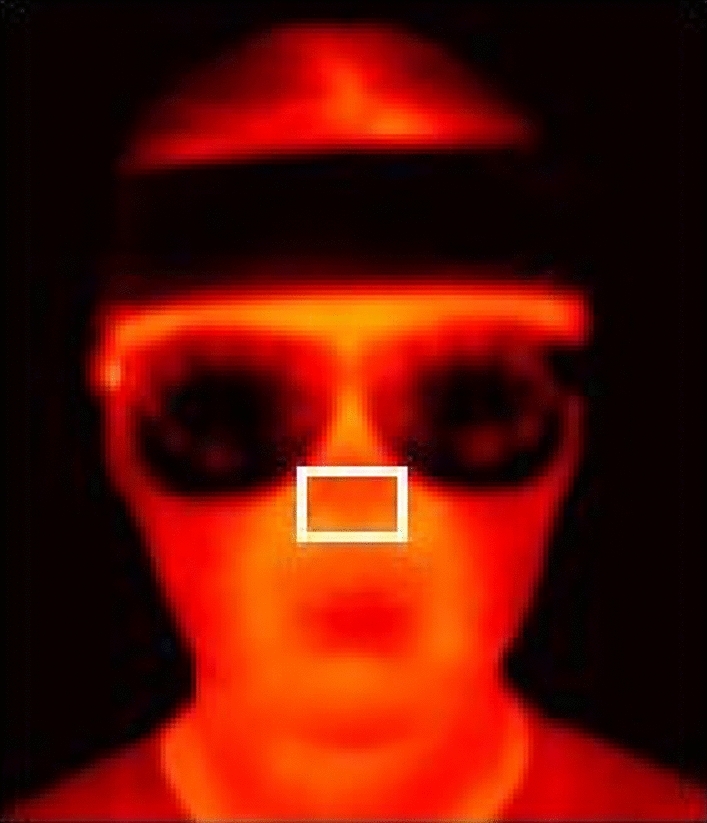
Extracted nasal region with privacy blur. Depicts the tracked nasal region used for thermal analysis while maintaining participant anonymity.

Alterations in nasal tip temperature are recognized as reliable indicators of anxiety, with lower temperatures correlated with heightened anxiety levels^61–63^. Fig. 3 displays the extracted nose region from the thermal images, which were intentionally blurred to preserve privacy. We employed the open-source project TIPA^64^ to automatically track the nasal region of interest. The device was positioned 70 centimeters from the participants, with blackbody calibration conducted prior to recording. This setup allowed us to monitor participants’ nasal temperatures during the presentation task, potentially providing insights into their physiological responses.

**STAI-S** The STAI^65^ consists of two scales: the State Anxiety Scale (STAI-S) and the Trait Anxiety Scale (STAI-T). The STAI-S assesses an individual’s feelings and emotions at the present moment, whereas the STAI-T measures more general and enduring anxiety tendencies. For our experiment, we utilized the Japanese STAI-S (Form-JYZ), which comprises 20 items (e.g., “I feel tense”) that are presented in a 4-point Likert scale format. The total score for state anxiety ranges from 20 to 80. Lower scores (20-44) indicate lower levels of state anxiety, reflecting calmness or a lack of temporary anxiety. Moderate scores (45-54) suggest moderate levels of state anxiety. Higher scores (55-80) indicate higher levels of state anxiety, reflecting greater temporary anxiety. After completing the presentation task, participants self-reported their current emotional state using the STAI-S. Previous studies have demonstrated strong internal consistency for the STAI-S^66,67^. In our study, the STAI-S exhibited exceptional internal consistency, with $\alpha$=.95.

**PSP**  The PSP^68^ evaluates speech performance using 17 items, which cover both general aspects (5 items, e.g., “Appeared Nervous”) and specific aspects (12 items, e.g., “Voice Quivered”) of the speech. The participants were asked to rate their agreement with various statements regarding their speech performance on a scale ranging from 0 (“not at all”) to 4 (“very much”). Previous studies have consistently shown strong internal consistency when using the PSP scale for self-evaluation^67,69–71^. In our current study, the internal consistency of the total scale score for participant ratings is excellent, with $\alpha$=.87.

**Self-designed questionnaire** We designed an 8-item questionnaire using a 5-point Likert scale (items are listed in Table 1), with response options ranging from 1 (strongly disagree) to 5 (strongly agree). The purpose of this questionnaire was to evaluate the extent to which various factors potentially influencing anxiety levels were effectively controlled during the experiment. Rather than measuring a single latent construct, the questionnaire captured a broad range of participants’ subjective experiences across multiple dimensions, including perceived familiarity, perceived kindness, and distraction. Additionally, an open-ended section was included to allow participants to freely share their thoughts and feelings, providing further qualitative insights.

**Table 1:** Self-designed questionnaire items.

1. The topic of the speech is difficult.	
2. I am accustomed to video interviews.	
3. I am familiar with the face of the interviewer.	
4. The interviewer was kind.	
5. I have a strong desire to speak to the interviewer.	
6. The expression of the interviewer is stern.	
7. The face of the interviewer is distracting.	
8. The fear of interviews is alleviated.	

### Experiment procedure

**Participants** We recruited participants by sending an invitation email to all master’s and doctoral students at our university. The email outlined the experiment content, which is a real video interview experiment where participants deliver a presentation to an interviewer who uses a facial filter. A total of 45 participants, consisting of 33 males and 12 females, aged 22 to 29 years, were evenly distributed across three groups. Participants assigned to the GFI group were informed of submitting a frontal photo of a good friend. While the sample size of 45 participants may appear modest for a between-subjects design involving three conditions, it aligns with standards in the human-computer interaction (HCI) field. Based on a survey by Caine^72^, the average number of participants per condition in CHI publications is approximately 13. Our study exceeds this benchmark with 15 participants per condition, providing reasonable confidence in the robustness of the findings within this research context.

Considering that the interview language in the region was primarily Japanese, Japanese was designated as the language for oral presentations in the experiment. Among the participants, nine were native Japanese speakers. The remaining non-native Japanese participants possessed a proficiency level of N2 or above on the Japanese Language Proficiency Test, a standardized assessment certifying non-native speakers’ Japanese language skills. A passing grade of N2 or above demonstrates strong abilities in comprehending complex texts, engaging in advanced conversations, and writing at a high level, making it widely recognized for non-native speakers working, studying, or living in Japan. We evenly distributed the participants’ Japanese proficiency levels across the three groups. All participants had experience in online Japanese-language interviews, whether for employment, academic advancement, or simulation training.

On the day of the experiment, participants were provided with a verbal introduction by the experimenter, following the details outlined in a written document. This introduction covered the purpose and content of the experiment, instructions on the types of equipment to be used, details about the data to be collected, its storage duration, and the compensation rate set by the university. They were also informed that the interviewer’s appearance in the video call had been modified using a facial filter and that behind the filter was a real person from outside the university who would evaluate their presentation. The experimenter also emphasized that participants could stop the experiment at any time if they felt uncomfortable. Once participants fully understood the provided information and had no further questions, they voluntarily signed a consent form and completed a personal information sheet to receive compensation.

Subsequently, we introduced a separate document containing screen transition demonstrations. This document included the content for the neutral reading task, simple diagrams representing the interviewer shown during the presentation task, and the quiet waiting instructions displayed at the end. We emphasized that any errors or omissions during the reading task would not affect the experiment, as the data collection focused on the presentation task. This served to highlight the importance of the presentation task while reducing potential pressure during the reading task. Participants were given five minutes to familiarize themselves with the reading task, which also included instructions to minimize head movement and avoid ending their presentation prematurely. Then, participants received a sheet of paper with the assigned speech topic and blank space to draft notes or prepare their presentations during the 7-minute preparation time.

**Fig. 4:**
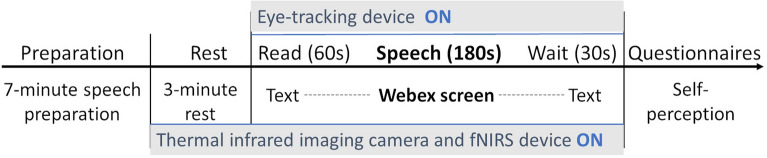
Overview of the experimental procedure. Illustrates task order and device usage during the presentation experiment.

**Fig. 5:**
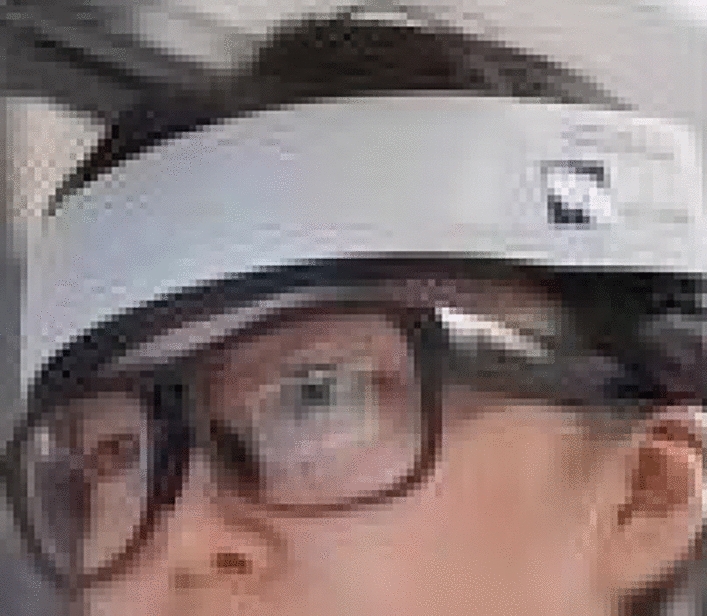
Participant wearing measurement devices. Displays the setup of eye-tracking glasses and the fNIRS device during data collection.

Afterward, participants were directed to sit in front of the computer screen. The experimenter replaced the necessary lenses and connected the head and recording units as outlined in the Tobii Pro Glasses 3 manual. Participants wore eye-tracking glasses continuously, even during rest periods, to adapt to their weight (76.5 grams, including the connecting cable). Participants then positioned the fNIRS device on their dry foreheads, ensuring no interference from hair, as per the Hot-2000 manual. Once the placement was confirmed, device measurements began. The experimenter also activated the infrared camera at this stage. After a 3-minute rest, the experimenter calibrated the eye-tracking glasses according to the Tobii Pro Glasses 3 User Manual. During calibration, a calibration card was placed on the computer screen, and participants were instructed to focus on its center. Calibration was initiated via the application interface, and upon successful completion, eye-tracking data recording commenced. Figure 4 illustrates the operational status of these devices during the experimental process, while Fig. 5 presents a photo of a participant wearing both the eye-tracking and fNIRS devices. Privacy concerns were addressed by applying image blurring to the photo.

After calibration, the experimenter left the room, and the computer screen displayed the reading task. Participants independently completed the following tasks shown on the screen. The room temperature was controlled between 18-$20^{\circ }\text {C}$, which was considered a comfortable temperature by the participants. The interviewer was located in a room on the other side of the campus, and his side of the video call was set to mute to act as the audience. As a result, participants were in a quiet room while delivering the presentation to the interviewer.

After 250 seconds, the experimenter returned and shut down the types of equipment. Participants then completed the questionnaires on the computer screen using a mouse. In the final section of the self-made questionnaire, they were encouraged to verbally describe their experiences and express their feelings. The experimenter recorded their responses through audio. Finally, the experimenter engaged in a brief conversation with them to address any questions they had. The entire experiment lasted approximately 40 minutes, and participants received a compensation of 1,000 JPY.

**Ethics and Consent Statement** The study was approved (Approved No. 04-013) by the Japan Advanced Institute of Science and Technology Life Science Committee on July 27, 2022. All methods were performed in accordance with the relevant guidelines and regulations. The recruitment period for the validation experiment was from August 1, 2022, to November 1, 2022. Participation was voluntary and signed written informed consent forms. Additionally, participants were informed that they could withdraw or terminate the experiment at any time without any negative consequences. Pre-counseling and post-counseling sessions were conducted before and after the experiment, respectively, to alleviate potential discomfort caused by the measurement devices and to mitigate the potential impact of the experiment.

All figures containing identifying information in this manuscript were obtained with the informed consent of the respective participants for the publication of their identifying information/images in the online open-access publication.

### Experiment results

Due to technical issues, three participants required additional data and were subsequently excluded from the analysis. Thus, the statistical analysis included data from 45 participants, consisting of 33 males and 12 females between the ages of 22 and 29.

Because the data were not normally distributed (Shapiro-Wilk, p<.05), we employed non-parametric tests for the statistical analysis. For each dependent variable, we performed a Kruskal-Wallis omnibus test followed by Mann-Whitney U tests for pairwise group comparisons when appropriate, and Bonferroni correction for multiple comparisons was applied as implemented in IBM SPSS Statistics version 27 (https://www.ibm.com/products/spss-statistics). However, correction was not applied across the full set of all *p* values reported in the study.

#### Perception

**Fig. 6:**
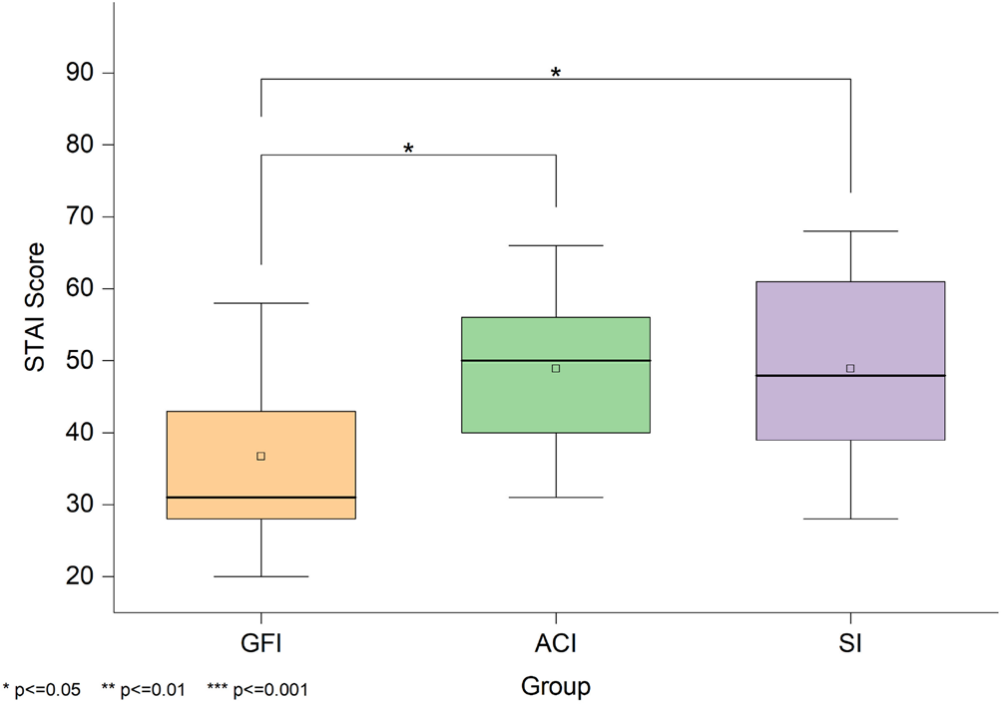
State-Trait Anxiety Inventory-State (STAI-S) scores across the three groups. Shows anxiety level differences among the Good Friends Interviewer (GFI), Anime Character Interviewer (ACI), and Stranger Interviewer (SI) groups, with lower scores indicating lower anxiety.

**STAI-S** Figure 6 shows the distribution of scores on the self-reported current anxiety state scale among the three groups, with higher scores indicating higher anxiety levels. A Kruskal-Wallis test revealed a significant difference in state anxiety scores across the three groups, H(2)=7.81, *p*=.020. The median scores were 31 for GFI, 50 for ACI, and 48 for SI. Follow-up Mann-Whitney U tests with Bonferroni correction showed that the GFI group reported significantly lower anxiety than both the SI group (*Z*=-2.41, adjusted *p*=.048, *r*=.36) and the ACI group (*Z*=-2.43, adjusted *p*=.045, *r*=.36). No significant difference was observed between the ACI and SI groups (*Z*=.03, adjusted *p*=1, *r*=0).

**Fig. 7:**
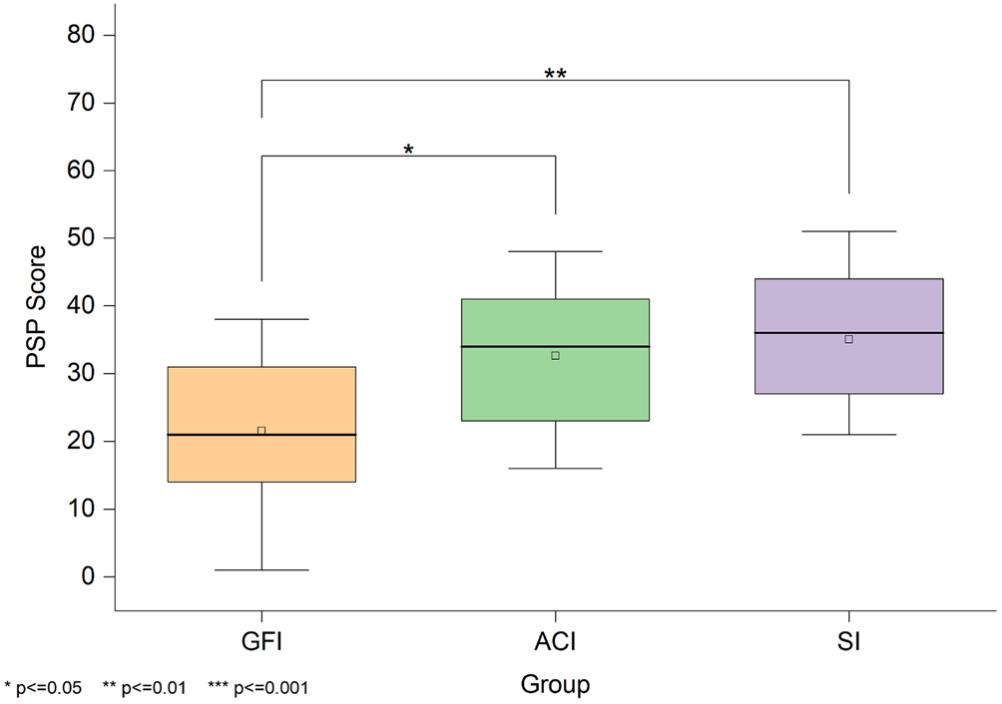
Perception of Speech Performance (PSP) scores across the three groups. Shows perceived speech performance in the Good Friends Interviewer (GFI), Anime Character Interviewer (ACI), and Stranger Interviewer (SI) groups, where lower scores correspond to better self-assessed performance.

**PSP** Figure 7 shows the PSP scores of the participants’ self-evaluations in the three groups, with higher scores indicating a poorer perceived performance. A Kruskal-Wallis test revealed a significant difference in self-perceived performance across the three groups, H(2)=10.72, *p*=.005. The median scores were 21 for GFI, 34 for ACI, and 36 for SI. Post hoc Mann-Whitney U tests with Bonferroni correction indicated that participants in the GFI group rated their performance significantly better than those in the SI group (*Z*=-3.07, adjusted *p*=.006, *r*=.46) and the ACI group (*Z*=-2.53, adjusted *p*=.035, *r*=.38). No significant difference was found between the ACI and SI groups (*Z*=-.54, adjusted *p*=1, *r*=.08).

**Self-designed questionnaire** Item 3 – Familiarity with the Interviewer’s Face. A Kruskal-Wallis test revealed a significant group difference in perceived familiarity with the interviewer’s face, H(2)=29.45, $p<$.001. Follow-up Mann-Whitney U tests with Bonferroni correction showed that participants in the GFI group rated the interviewer as significantly more familiar than both the SI group (*Z*=4.23, adjusted $p<.001$, *r*=.63) and the ACI group (*Z*=5.06, adjusted $p<.001$, *r*=.75). No significant difference was found between the ACI and SI groups (*Z*=-.83, adjusted *p*=1, *r*=.12).

Item 4 – Perceived Kindness of the Interviewer. A Kruskal-Wallis test indicated a significant difference in perceived kindness across groups, H(2)=8.06, *p*=.018. Post hoc comparisons revealed that participants in the GFI group perceived the interviewer as significantly kinder than those in the ACI group (*Z*=2.67, adjusted *p*=.023, *r*=.40). No statistically significant differences were observed between GFI and SI (*Z*=2.18, adjusted *p*=.088, *r*=.32) or between ACI and SI (*Z*=-.49, adjusted *p*=1.00, *r*=.07).

Item 8 – Fear Reduction During the Interview. A Kruskal-Wallis test found a significant difference in interview-related fear reduction, H(2)=9.45, *p*=.009. Pairwise tests indicated that the GFI group reported significantly more reduction in fear compared to the SI group (*Z*=3.06, adjusted *p*=.007, *r*=.46), while no significant differences were found between GFI and ACI (*Z*=1.76, adjusted *p*=.236, *r*=.26) or between ACI and SI (*Z*=1.31, adjusted *p*=.575, *r*=.20). No significant differences were observed between groups for the other items.

Several noteworthy points emerged from the spontaneous expressions of the participants. In the GFI group, participants mentioned experiencing dissonance when seeing foreign expressions or angles shown on familiar faces. In the ACI group, many participants expressed difficulty receiving feedback from the anime character, which caused unease. In the SI group, some participants reported that the interviewer was familiar because it aligned with their expectations of an interviewer, despite never having met him in real life.

#### Behavior

**Fig. 8:**
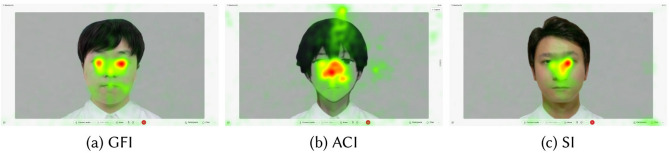
Overlaid heat maps of gaze fixation during the presentation task. Shows the superimposed gaze patterns from the participants under the Good Friends Interviewer (GFI), Anime Character Interviewer (ACI), and Stranger Interviewer (SI) groups during presentations. Colors from green to yellow to red indicate fixation durations from short to long.

**Eye-contact** The recorded data were analyzed using Tobii Pro Lab version 1.207 (https://www.tobii.com/products/software/ behavior-research-software/tobii-pro-lab) to identify and quantify gaze interactions. We focused on the area of interest (AOI) around the interviewer’s eyes and analyzed the participants’ gaze duration and frequency within this area. To accurately capture participants’ gazes in dynamic situations, we utilized a dynamic AOI that can be repositioned to match targets in the video. We employed an automatic mapping system within the software to map the positions of an AOI from dynamic videos to static photos. Subsequently, frame-by-frame visual inspection and adjustments were made to ensure accurate correspondence. To ensure consistent exposure to the interviewer’s gaze, we analyzed the first 120 seconds of the presentation task; two participants finished their speech slightly over two minutes, and the interviewer maintained a downward gaze during the remaining time. We applied the Tobii I-VT attention filter with a threshold of 100 degrees per second, calculating both the duration and frequency of the participants’ gazes in the dynamic AOI. The heat maps in Fig. 8 depict the overall distribution of the eye movement data for each group. In the GFI, we focused the data on the specific photo, which is shown in a). Images a), b), and c) are actual screenshots of the screen participants faced during the presentation task. The gradient from green to yellow to red represents the continuum of fixation duration, which ranges from short to long.

**Fig. 9:**
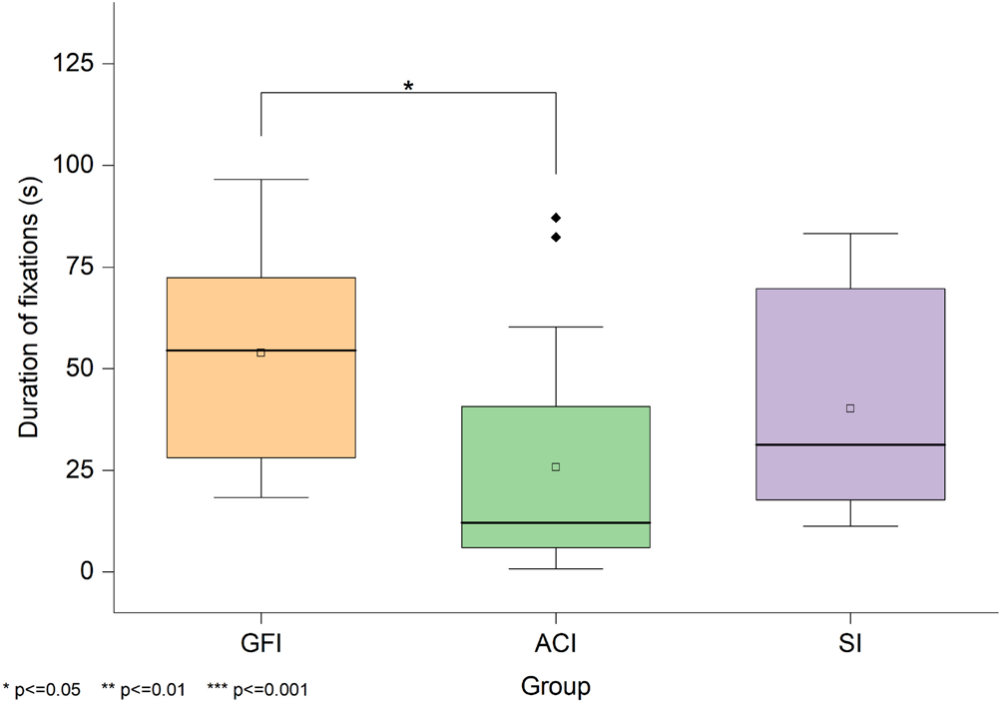
Fixation duration within the eye region of interest. Shows the total time participants spent fixating on the interviewer’s eye region under the Good Friends Interviewer (GFI), Anime Character Interviewer (ACI), and Stranger Interviewer (SI) groups.

The total duration of the whole fixation within the AOI under the three groups is shown in Fig. 9. A Kruskal-Wallis test revealed a significant difference in the total duration of fixations on the interviewer’s eyes across the three groups, H(2)=9.15, *p*=.010. The median scores were 54.53 for GFI, 12.10 for ACI, and 31.33 for SI. Follow-up Mann-Whitney U tests with Bonferroni correction showed that participants in the GFI group maintained significantly longer gaze duration than those in the ACI group (*Z*=3.01, adjusted *p*=.015, *r*=.45). No significant differences were observed between GFI and SI (*Z*=1.22, adjusted *p*=1.00, *r*=.18) or between ACI and SI (*Z*=-1.72, adjusted *p*=.516, *r*=.26).

**Fig. 10:**
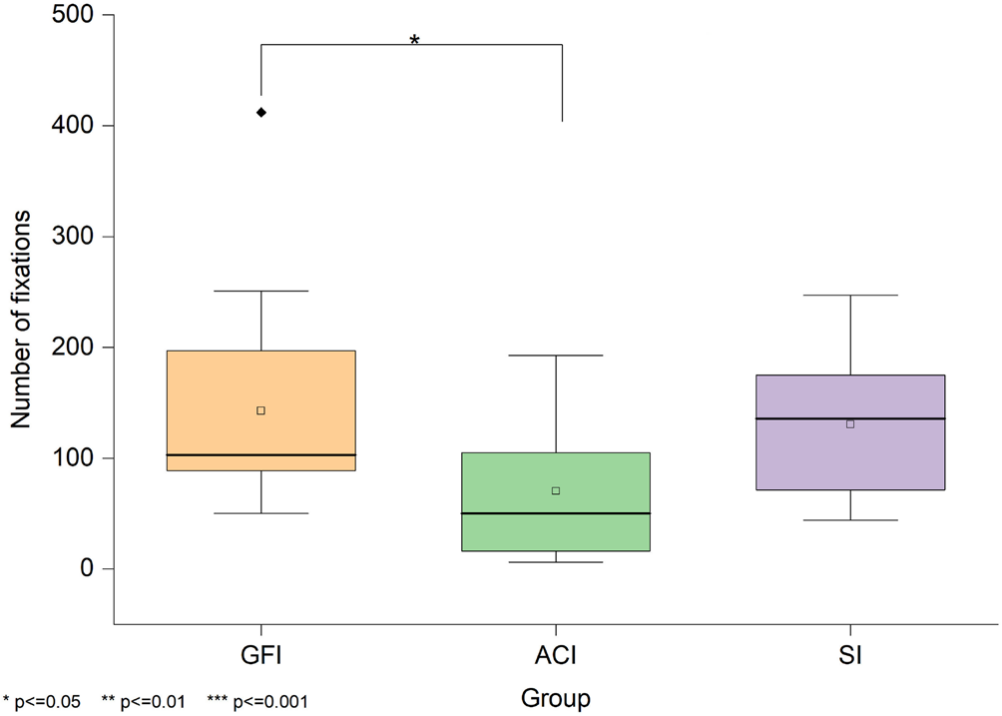
Fixation count within the eye region during the presentation task. Shows the total number of fixations on the interviewer’s eye region across the Good Friends Interviewer (GFI), Anime Character Interviewer (ACI), and Stranger Interviewer (SI) groups.

The number of whole fixations within the AOI under the three groups is shown in Fig. 10. A Kruskal-Wallis test indicated a significant difference in the number of fixations across conditions, H(2)=8.68, p=.013. The median scores were 103 for GFI, 50 for ACI, and 136 for SI. Follow-up comparisons indicated that participants in the GFI group made significantly more fixations on the interviewer than those in the ACI group (*Z*=2.69, adjusted *p*=.043, *r*=.40). No significant differences were found between the GFI and SI groups (*Z*=.22, adjusted *p*=1.00, *r*=.03) or between the ACI and SI groups (*Z*=-2.38, adjusted *p*=.103, *r*=.36).

**Word count** We analyzed the number of complete words spoken by the participants during the 3-minute interview sessions. We employed Whisper software (https://github.com/openai/whisper) to transcribe the participants’ spoken content into text and subsequently quantified it. A Kruskal-Wallis test revealed a significant difference in word count across the three groups, H(2)=6.06, *p*=.048. The median scores were 517 for GFI, 512 for ACI, and 441 for SI. Post hoc comparisons indicated only a trend toward a higher word count in the GFI group compared to the SI group (*Z*=2.43, adjusted *p*=.090, *r*=.36).

#### Correlation

We utilized Spearman correlation analysis to individually compare the following variables in pairs: STAI_S, PSP, fixation duration, fixation number, and word count (see Table 2). The results revealed a strong positive correlation between fixation duration and fixation number. Additionally, a strong positive correlation was observed between the STAI_S and PSP scales, indicating that higher self-reported anxiety levels were associated with poorer perceived performance. Both scales displayed a weak negative correlation with word count, suggesting that speaking less during the presentation was somewhat associated with higher self-perceived anxiety and lower self-perceived performance.

**Table 2:** Correlations among variables in the validation experiment.

	STAI_S	PSP	FD	FN	Word_count
STAI_S					
Spearman correlation	1	$$0.83^{**}$$	-0.111	-0.040	$$-0.301^{*}$$
Sig. (2-tailed)		0.001	0.467	0.793	0.044
PSP					
Spearman correlation	$$0.83^{**}$$	1	-0.143	-0.058	$$-0.352^{*}$$
Sig. (2-tailed)	0.001		0.347	0.705	0.018
Fixation_duration (FD)					
Spearman correlation	-0.111	-0.143	1	$$0.792^{**}$$	0.046
Sig. (2-tailed)	0.467	0.347		0.001	0.763
Fixation_number (FN)					
Spearman correlation	-0.040	-0.058	$$0.792^{**}$$	1	-0.013
Sig. (2-tailed)	0.793	0.705	0.001		0.932
Word_count					
Spearman correlation	$-0.301^{*}$	$-0.352^{*}$	0.046	-0.013	1
Sig. (2-tailed)	0.044	0.018	0.763	0.932	

$^{*}$ Correlation is significant at the 0.05 level (2-tailed).

$$^{**}$$ Correlation is significant at the 0.01 level (2-tailed)

#### Physiological data

**Nasal temperature** To minimize individual differences, we first calculated the nasal temperature changes by subtracting the temperature recorded during a continuous 120-second resting period from that recorded during the first 120-second presentation task period, using the same rationale as in the eye-contact analysis. To capture the psychological changes triggered by the gaze of the interviewer, we further subtracted the average nasal tip temperature from the last 30 seconds of the reading task. The last 30 seconds of the reading task were selected to minimize the influence of the experimenter’s presence, as participants were alone in the room during this period after the experimenter had left, ensuring a consistent environment for baseline recording.

**Fig. 11:**
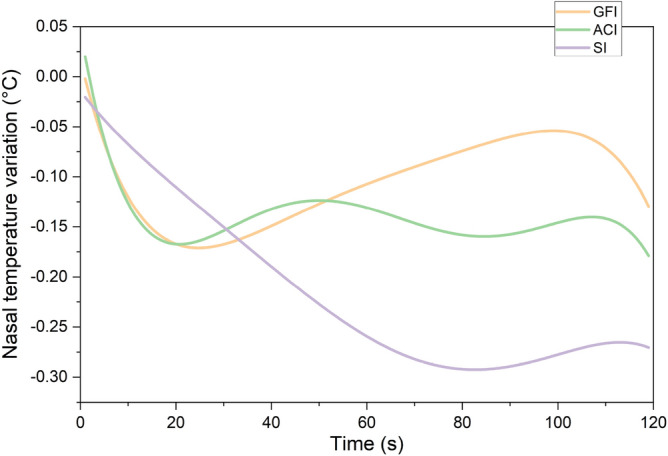
Group-averaged nasal temperature changes. Shows nasal temperature changes during presentations under the Good Friends Interviewer (GFI), Anime Character Interviewer (ACI), and Stranger Interviewer (SI) groups as a physiological indicator of anxiety, with lower temperatures correlated with heightened anxiety levels.

As shown in Fig. 11, which displays the average nasal temperature variations across participants for each group during the presentation task, the SI group appears to exhibit a more substantial decrease, while the other two groups show little difference. However, statistical analysis did not reveal any significant differences in nasal temperature changes among the three groups during this period.

**Fig. 12:**
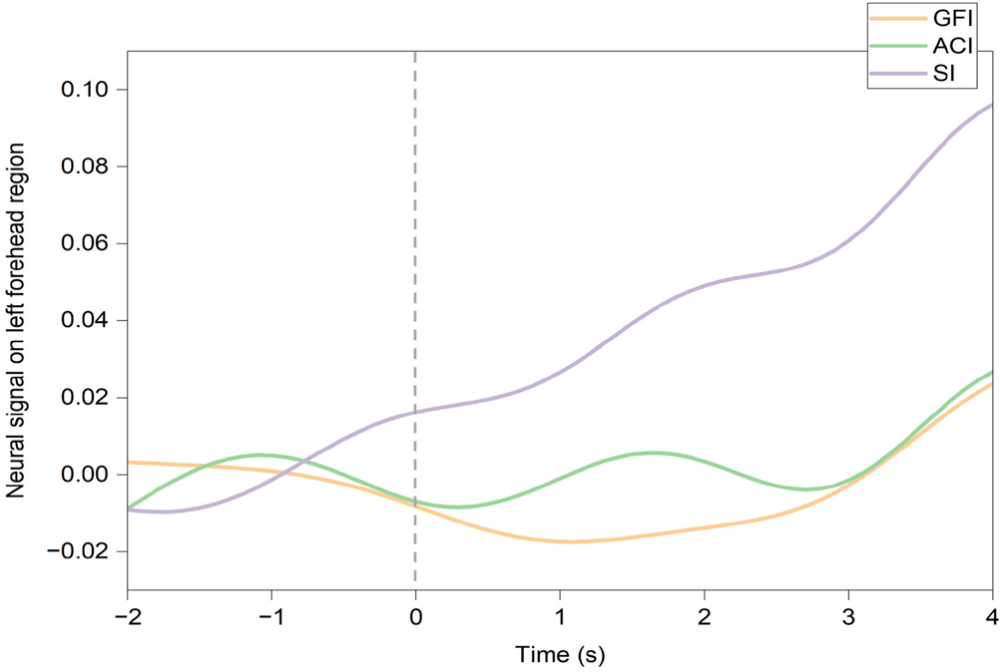
Group-averaged changes in left prefrontal cerebral blood flow. Shows changes in left prefrontal cortical blood flow at the time of interviewer appearance across the Good Friends Interviewer (GFI), Anime Character Interviewer (ACI), and Stranger Interviewer (SI) groups, reflecting potential anxiety-related neural responses.

**Brain activity** We calculated the changes in cerebral blood flow between -2 and 4 seconds from the start of the interviewer-interviewee meeting, aligning with the observation time in the previous study^73^. However, no significant differences were observed across conditions. Figure 12 illustrates the mean change in cerebral blood flow over time in the left PFC. The right side of the dashed line corresponds to the change in cerebral blood flow after the interviewer’s appearance, corrected by the mean value 2 seconds before the interviewer’s appearance (left side of the dashed line). In our experiment, the observed increase in cerebral blood flow within the left PFC in the SI group could be related to the modulation of anxiety states experienced when facing the audience.

## Discussion

With the mainstream adoption of video conferencing, an increasing number of studies have found that the anxiety experienced during remote interactions can be comparable to that of face-to-face interactions^37,74^. This is especially true for individuals with high social anxiety, who may even experience heightened anxiety in video conferences^47^. As video conferencing continues to play a critical role in academic, professional, and interpersonal communication, developing strategies to reduce associated anxiety becomes increasingly important^34^.

The findings show that participants who saw their interviewer as a familiar friend (GFI) reported lower anxiety and better self-evaluation than those who saw either a stranger (SI) or an anime character (ACI). Additionally, participants in the GFI condition exhibited more gaze behaviors toward the interviewer compared to those in the ACI condition and demonstrated a greater tendency to produce more verbal output than those in the SI condition. These patterns offer insight into how perceived social context and emotional cues shape user experience in virtual settings, highlighting the potential of leveraging familiar and emotionally resonant visual representations to foster more comfortable and engaged interactions in video-mediated communication.

### Familiarity promotes a sense of psychological safety

The GFI condition consistently showed more favorable outcomes across subjective measures. This is consistent with prior work suggesting that familiarity reduces social-evaluative threat by activating schemas of trust, acceptance, and prior positive experience^36,75^. The friend’s face may have served as a cognitive anchor, shifting the user’s interpretation of the situation from formal evaluation to informal support. This may explain why participants felt more relaxed and rated their performance more positively—even though the facial expression itself was neutral.

Importantly, these findings extend previous work on face-to-face familiarity^76^ into virtual environments where the familiarity is artificially induced. These results also extend our understanding of how social meanings are actively constructed and preserved during interactions. According to Constructivist theory^77,78^, individuals interpret external stimuli through the lens of prior experiences and knowledge, dynamically shaping their perceptions in ways that align with their expectations and relational histories. In this study, when participants chose to believe they were interacting with a friend, they not only experienced reduced anxiety but also engaged in spontaneous meaning-making processes that preserved the social significance of facial identity. This suggests that the cognitive frameworks individuals bring into social interactions can, to some extent, shield them from the objective reality of the situation, allowing them to maintain emotional and relational continuity even when the interaction partner is known to be non-authentic. Although technical limitations during the experiment may have caused momentary confusion due to changes in facial angles, these disruptions did not fundamentally undermine participants’ ability to preserve the social and emotional meanings attached to the interaction. Such findings are consistent with previous research demonstrating that cognition is inherently situated within social contexts and interactions^79^, highlighting the flexibility of the mind in sustaining social connections through constructive processes. Moreover, these results imply that interventions leveraging perceived social presence could effectively reduce anxiety in contexts where in-person interaction is not feasible, such as online therapy or remote interviews.

### Stylized avatars may disrupt social interpretation

In contrast, replacing the interviewer’s face with an anime character failed to reduce anxiety or improve self-evaluation. The ACI condition elicited the least visual engagement (shorter and fewer eye fixations), despite being designed to be visually appealing. This suggests that aesthetic appeal alone is insufficient to foster comfort in evaluative contexts.

One possible explanation lies in the mismatch between the avatar’s expressive capacity and participants’ expectations. While anime characters are often associated with warmth and expressiveness^37^, the avatar used in this study had a neutral or slightly stern expression, coupled with formal attire. This inconsistency may have produced a subtle dissonance or expectation violation^80^, undermining the avatar’s intended supportive function. Further, the simplified and stylized facial features likely limited participants’ ability to interpret emotional nuance^81^, reducing the effectiveness of social feedback. In high-anxiety situations, the ability to detect affirming or disapproving cues becomes critical^76^, and the absence of such signals may increase uncertainty. The large eyes of the character, a common stylized feature, may have amplified the sense of being watched, contributing to gaze aversion—a typical anxiety response^82^.

While the avatar was designed to provide a non-threatening social presence, its limited dynamic expressiveness may have constrained its capacity to modulate participants’ anxiety effectively. Additionally, the static or limited gaze behavior of the avatar may have been interpreted as staring, which, in socially anxious individuals, can be perceived as threatening or evaluative, thereby inadvertently increasing tension rather than reducing it. Taken together, these factors highlight the complexity of using stylized avatars as social buffers, indicating that careful calibration of appearance, expressiveness, and context-specific behaviors is critical to achieving the desired psychological outcomes in high-stress environments.

### Anxiety and behavioral correlates

Our correlational analysis revealed a strong positive association between self-reported state anxiety and poorer perceived performance, consistent with prior findings^83^. Individuals experiencing higher levels of state anxiety tend to interpret their performance more pessimistically, potentially due to heightened attentional focus on perceived mistakes or failures. This pessimistic interpretation may reduce their sense of accomplishment, which in turn can undermine their engagement and self-efficacy in future tasks.

Additionally, we observed a weak negative correlation between anxiety and word count, aligning with earlier findings that anxiety may reduce verbal output in public speaking situations^46,84,85^. Heightened anxiety is likely to increase cognitive load, which can restrict the cognitive resources available for speech planning and production, leading to reduced verbal output during presentation tasks.

Interestingly, although gaze behavior (fixation metrics) varied significantly between groups, it did not directly correlate with STAI-S scores. This may reflect the unique dynamics of video conferencing environments, where maintaining eye contact or gaze direction carries different social significance and cognitive demands compared to in-person interactions^32^. In virtual settings, factors such as screen layout, camera positioning, and the absence of direct physical presence may decouple gaze behaviors from subjective anxiety levels, suggesting that gaze metrics alone may not serve as reliable indicators of state anxiety in remote communication contexts.

### Implications for design of virtual interactions

Our findings suggest that familiarity—even when artificially induced—can serve as a powerful psychological buffer in high-stressful video communication. The mere appearance of a trusted individual may trigger positive emotional associations and reduce anticipatory anxiety, thereby facilitating a more adaptive emotional state during the task. These results highlight the importance of social context design in virtual platforms, where the interface can be customized to shape user experience and performance.

Conversely, the limited effect of the anime character on reducing anxiety suggests that anthropomorphic design alone is not universally effective. One possible explanation is that such stylized representations may not evoke consistent emotional resonance across individuals. Emotional comfort in social interactions often depends not only on visual aesthetics but also on personal associations and affective bonds^34^. For individuals lacking a strong connection to anime culture, the character may have felt unfamiliar or even distanced, thereby failing to provide a sense of psychological safety. This highlights the importance of allowing users to select or customize avatars that reflect their own preferences or emotional anchors.

### Limitations and future directions

Several limitations of this study should be acknowledged.

First, the sample size of 45 participants, while consistent with standards in similar HCI studies^72^, limits statistical power in a three-group between-subjects design. Future studies with larger and more diverse samples are needed to validate and generalize the current findings.

Second, our self-designed questionnaire was intended to assess distinct subjective dimensions—such as familiarity, friendliness, and fear—rather than a single latent construct. Due to the conceptual heterogeneity of these items, we did not compute a composite score or perform psychometric analyses (e.g., Cronbach’s alpha). While this approach allowed us to examine specific experiential components, analyzing each item separately increases the family-wise error rate and the possibility of Type I errors. Accordingly, these questionnaire results should be interpreted as exploratory and supplementary.

Third, although Bonferroni correction was applied within each set of planned comparisons, and the adjusted *p* values under SPSS tend to be more stringent, the absence of a global correction across all statistical tests in the study remains a limitation.

Fourth, participants were given the option to discuss either their strengths, weaknesses, or both. While this was intended to promote comfort and reduce presentation avoidance, it may have inadvertently introduced a confounding variable. Future studies should consider controlling topic content to ensure more uniform cognitive and emotional demands across participants.

Fifth, although Japanese language proficiency was controlled, other factors such as topic familiarity, prior interview experience, and trait anxiety were not measured. These individual differences may interact with anxiety in complex ways^36,83,86^ and merit closer attention in future work. Additionally, the gender of the interviewer as presented via the facial filters was not standardized across conditions. Participants in the GFI group may have seen male or female interviewers, whereas SI and ACI were limited to male-presenting avatars. Gender composition can influence social dynamics and anxiety^87^, and should be balanced in subsequent designs.

Sixth, physiological data (infrared thermography and fNIRS) did not reveal significant group differences. This may be due to single-trial design, equipment limitations, or insufficient signal sensitivity. More robust physiological measures and multi-trial designs may yield stronger effects^59^.

Finally, due to technical constraints, real-time audience masking in this study was implemented by the interviewer rather than being controlled by the participants themselves. While this ensured standardization across sessions, it limited the autonomy of participants in managing their own anxiety during evaluative interactions.

In the future, under strict adherence to robust ethical principles^37^, it would be valuable to develop systems that allow individuals who are more prone to experiencing anxiety to apply such facial filters during interactions without requiring disclosure to the other party. For example, when designing software, users can be prompted upon first entry to choose whether they consent to having face filters applied to them by others during interactions. Once the user has given consent, they will not receive further notifications when others apply these filters.

Such systems could empower users to engage in social, educational, or professional interactions with greater confidence, particularly in high-stakes situations such as interviews or public speaking, where visual self-presentation can be a significant source of stress. By giving users the ability to selectively manage the visual cues they receive, these tools could reduce cognitive load associated with self-monitoring and fear of negative evaluation, thereby supporting smoother and more effective communication.

Additionally, from a design perspective, the integration of customizable audience masking should be accompanied by clear, transparent consent protocols and opt-out options to ensure that all parties retain agency over their interaction experience. Such protocols not only uphold individual autonomy but also help set clear expectations within social and professional interactions, reducing potential misunderstandings or discomfort. Future research should systematically explore the psychological and interpersonal impacts of these user-controlled filtering systems, including their potential effects on trust, authenticity, relational dynamics, and perceptions of social presence. Understanding these impacts is crucial for identifying both the benefits and unintended consequences of audience masking in diverse contexts. Insights from this research could inform the development of ethically grounded, user-centered technologies that address emotional and accessibility needs while preserving the integrity of interpersonal communication in both remote and hybrid settings.

### Conclusion

This study provides initial empirical evidence that modifying the visual representation of an interviewer can affect users’ subjective experience in video calls. Specifically, presenting the audience as a good friend was associated with lower anxiety and higher self-rated performance. In contrast, the anime character filter, though theoretically promising, failed to produce the same benefit—possibly due to reduced emotional expressiveness and mismatched expectations. These findings highlight the potential of visual customization for anxiety reduction, while also underscoring the need for careful design considerations. Future work should further explore adaptive, personalized, and real-time interventions for supporting user comfort in digital communication.

## Data Availability

The datasets generated during/or analyzed during the current study are not publicly available due to portrait and privacy concerns, but they are available from the corresponding author upon reasonable request.
